# Complete Genome Sequence of a SARS-CoV-2 Strain Sampled in Morocco in May 2020, Obtained Using Sanger Sequencing

**DOI:** 10.1128/MRA.00387-21

**Published:** 2021-05-20

**Authors:** Abderrazak Rfaki, Nadia Touil, Mouhssine Hemlali, Sanaâ Alaoui Amine, Marouane Melloul, Moulay Abdelaziz El Alaoui, Hicham Elannaz, Amine Idriss Lahlou, Mostafa Elouennass, Khalid Ennibi, Elmostafa El Fahime

**Affiliations:** a Molecular Biology and Functional Genomics Platform, National Center for Scientific and Technical Research (CNRST), Rabat, Morocco; b Genomic Center for Human Pathologies (GENOPATH), Faculty of Medicine and Pharmacy, University Mohammed V, Rabat, Morocco; c Unité de Culture Cellulaire, CVMIT, HMI Med V, Rabat, Morocco; d Laboratoire de Botanique et de Protection des Plantes, UFR de Mycologie, Département de Biologie, Faculté des Sciences, Université Ibn Tofail, Kenitra, Morocco; DOE Joint Genome Institute

## Abstract

The complete genome sequence of a severe acute respiratory syndrome coronavirus 2 (SARS-CoV-2) strain was obtained. The strain was isolated from a nasopharyngeal swab specimen from a female patient in Rabat, Morocco, with coronavirus disease 2019 (COVID-19). This strain belongs to clade 20A and has 12 mutations and 8 amino acid substitutions compared to the reference strain Wuhan/Hu-1/2019.

## ANNOUNCEMENT

Severe acute respiratory syndrome coronavirus 2 (SARS-CoV-2), a member of the *Coronaviridae* family and *Betacoronaviru*s genus, is the causative agent of the coronavirus disease 2019 (COVID-19) pandemic. As of April 2020, positive cases rose continually, reached a peak in November 2020, and then decreased gradually until now (http://www.covidmaroc.ma/pages/Accueilfr.aspx). Analysis of the SARS-CoV-2 genome is crucial for fully understanding its spread and evolution as well as for vaccine development (1). Currently, SARS-CoV-2 whole-genome sequences are often generated by next-generation sequencing (NGS) (2), because of the fast speed and parallelism of the standard NGS methods; however, Sanger sequencing can also provide high-quality genome sequences (3).

Here, we report the complete genome sequence of SARS-CoV-2 from a Moroccan patient, using Sanger sequencing technology, based on multiple amplified nucleic acid fragments, according to CDC (4) protocol.

Strain SARS-CoV-2/human/MAR/Morocco_HMIMV_279CC/2020 was recovered from a nasopharyngeal swab specimen from a 48-year-old female patient in Rabat, Morocco, with COVID-19 on 10 May 2020.

Viral RNA was extracted using a MagPurix instrument (Zinexts Life Science, UK) and quality controlled using a NanoDrop spectrophotometer (Thermo Fisher Scientific, Germany). cDNA was generated using a Tetro cDNA synthesis kit (Bioline, UK), and real-time PCR (RT-PCR) was performed using MyFi mix (Bioline) according to the manufacturer’s protocol.

The whole-genome sequence of strain SARS-CoV-2/human/MAR/Morocco_HMIMV_279CC/2020 was generated using 38 pairs of specific primers covering the whole virus genome using a previously reported method (4). The PCR amplification yielded 38 amplicons, which were confirmed by gel electrophoresis analysis (1% agarose) and purified using an ExoSAP-IT PCR cleanup kit (Applied Biosystems, USA). The sequence reactions were performed using the BigDye terminator cycle sequencing kit v3.1 (Applied Biosystems), purified using the BigDye XTerminator purification kit (Applied Biosystems), and then loaded onto a SeqStudio genetic analyzer capillary sequencer (Applied Biosystems) according to the manufacturer’s instructions.

**FIG 1:**
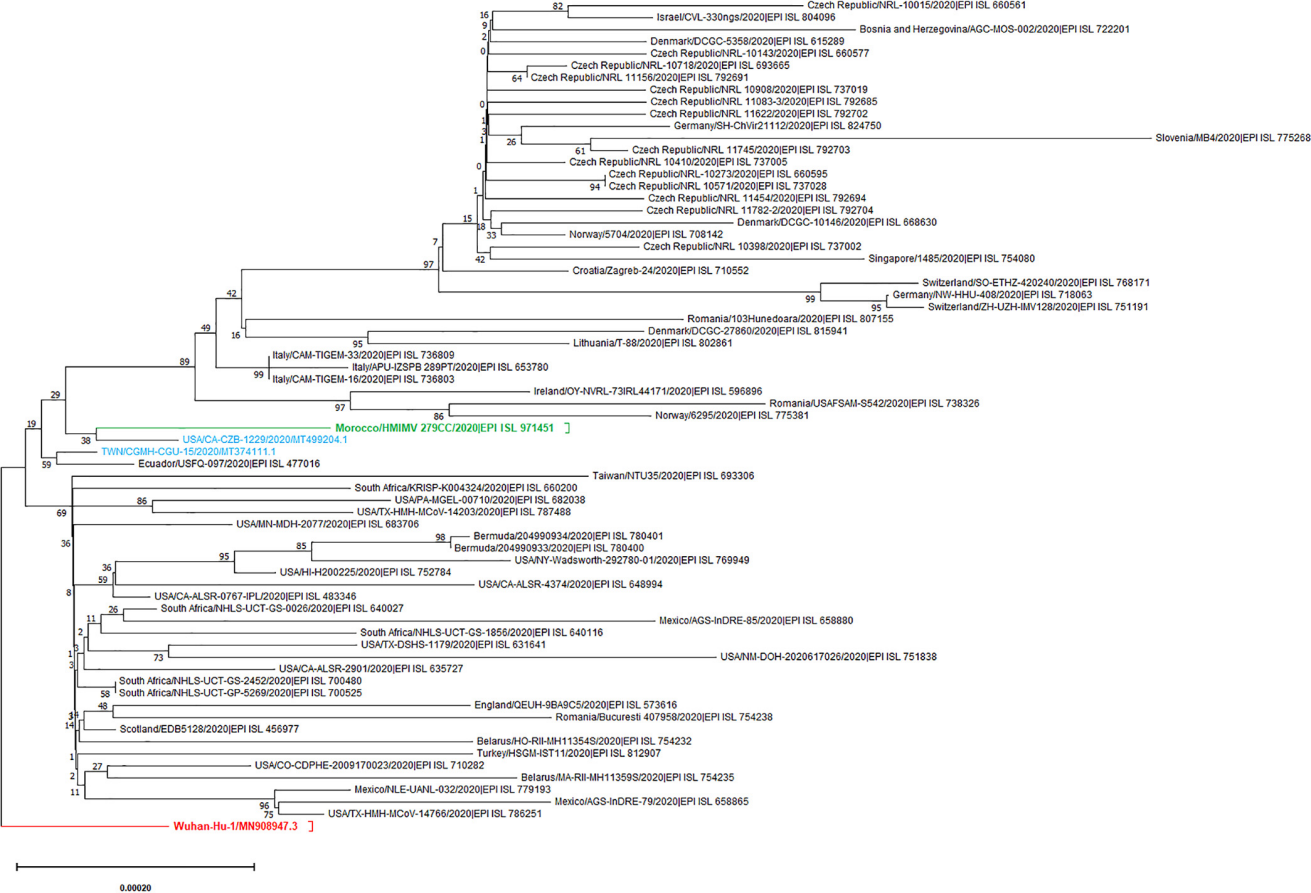
Phylogenetic tree of strain SARS-CoV-2/human/MAR/Morocco_HMIMV_279CC/2020. A total of 69 viral genome sequences are displayed, including (i) 66 genomes provided by Nextstrain (https://nextstrain.org/sars-cov-2/) on 10 February 2021, counting the sequence reported in this announcement (in green); (ii) 2 mostly similar genome sequences from NCBI, USA/CA-CZB-1229/2020 (GenBank accession number MT499204.1) and TWN/CGMH-CGU-15/2020 (MT374111.1) (in blue); and (iii) the reference genome of the SARS-CoV-2 Wuhan-Hu-1 isolate (MN908947.3) (in red). Multiple sequence alignments were conducted using MAFFT v7.221 (6) through the Galaxy platform (7), and the phylogenetic tree was constructed using MEGA X v10.2.0 (8) with the neighbor-joining algorithm with 1,000 bootstrap replicates. The tree structure was rooted in the position of reference strain Wuhan-Hu-1. Bar, number of nucleotide substitutions per site.

The obtained nucleotide sequences were evaluated, edited, and assembled, based on mapping to the reference sequence of the SARS-CoV-2 Wuhan-Hu-1 isolate (GenBank accession number MN908947.3) using Unipro UGENE software v37.0. The length of the final consensus sequence is 29,903 nucleotides with 37.96% GC content. NCBI BLASTN v2.11.0+ (5) showed that the genome was mostly similar (99.97%) to SARS-CoV-2/human/USA/CA-CZB-1229/2020 (MT499204.1) and to SARS-CoV-2/human/TWN/CGMH-CGU-15/2020 (MT374111.1), reported in the USA and Taiwan, respectively (Fig. 1).

Sequence quality control, clade assignment, and mutation determination were done using the Nextclade tool v0.12.0 (https://clades.nextstrain.org/). All tools were run using default parameters unless otherwise specified. The results showed that the SARS-CoV-2/human/MAR/Morocco_HMIMV_279CC/2020 genome belongs to clade 20A, with 12 mutations (A2568T, C3037T, C5884T, C8169T, C9907G, C14408T, C17104T, A20268G, G21795T, A23403G, G25563T, and G29734C) and 8 amino acid substitutions (ORF1a, Q768L; ORF1a, S2635L; ORF1a, Y3214; ORF1b, P314L; ORF1b, H1213Y; ORF3a, Q57H; S, R78M; and S, D614G), without amino acid deletions or PCR primer changes.

### Data availability.

The complete nucleotide sequence has been deposited in NCBI GenBank under accession number MW582699 and in the GISAID database under identifier EPI_ISL_971451.
